# Haplotype-resolved and near-T2T genome assembly of the African catfish (*Clarias gariepinus*)

**DOI:** 10.1038/s41597-024-03906-9

**Published:** 2024-10-07

**Authors:** Julien A. Nguinkal, Yedomon A. B. Zoclanclounon, Ronald M. Brunner, Yutang Chen, Tom Goldammer

**Affiliations:** 1 Research Institute for Farm Animals (FBN), Fish Genetics Unit, Dummerstorf, 18196 Germany; 2 Bernhard-Nocht Institute for Tropical Medicine, Department of Infectious Disease Epidemiology, Hamburg, 20359 Germany; 3 Plant Sciences and the Bioeconomy, Rothamsted Research, Harpenden, AL5 2JQ United Kingdom; 4 Molecular Plant Breeding, Institute of Agricultural Sciences, ETH Zurich, 8092 Zurich, Switzerland; 5 University of Rostock, Faculty of Agriculture and Environmental Sciences, Rostock, 18059 Germany

**Keywords:** Agricultural genetics, Genome assembly algorithms, Sequence annotation, Next-generation sequencing

## Abstract

Airbreathing catfish are stenohaline freshwater fish capable of withstanding various environmental conditions and farming practices, including breathing atmospheric oxygen. This unique ability has enabled them to thrive in semi-terrestrial habitats. However, the genomic mechanisms underlying their adaptation to adverse ecological environments remain largely unexplored, primarily due to the limited availability of high-quality genomic resources. Here, we present a haplotype-resolved and near telomere-to-telomere (T2T) genome assembly of the African catfish (*Clarias gariepinus*), utilizing Oxford Nanopore, PacBio HiFi, Illumina and Hi-C sequencing technologies. The primary assembly spans 969.62 Mb with only 47 contigs, achieving a contig N50 of 33.71 Mb. Terminal telomeric signals were detected in 22 of 47 contigs, suggesting T2T assembled chromosomes. BUSCO analysis confirmed gene space completeness of 99% against the *Actinopterygii* dataset, highlighting the high quality of the assembly. Genome annotation identified 25,655 protein-coding genes and estimated 43.94% genome-wide repetitive elements. This data provides valuable genomic resources to advance aquaculture practices and to explore the genomic underpinnings of the ecological resilience of airbreathing catfish and related teleosts.

## Background & Summary

The *Clariidae* family, commonly referred to as air-breathing catfish, constitutes a group of freshwater fish that can thrive out of water for extended periods of time by breathing oxygen from the atmosphere^1,2^. Some of these facultative air breathers have adapted to terrestrial life by developing the ability to survive in environments with low oxygen levels or stagnant water, such as mangrove swamps, muddy water, or flooded forests, which expand their access to new habitats and food sources^2,3^. According to FishBase resources^4^, the *Clariidae* family comprises 16 genera and 116 species, with clariids being the most widespread and diverse group with more than 32 recognized species. Many clariids are well-established aquaculture species, including African catfish (*Clarias gariepinus*, Burchell, 1822), one of Africa’s most promising endemic aquaculture fish^5^.

*C. garipinus* is found primarily throughout Africa, where it was first introduced in aquaculture around the mid-1970s. This omnivorous fish is quite resilient due to its ability to cope with extreme environmental conditions, tolerate various land-based farming practices and a large diet spectrum^6–8^. In addition to its rapid growth and extreme robustness, *C. gariepinus* can withstand high levels of ultraviolet B (UV-B) radiation and dramatic temperature fluctuations in non-aquatic environments^9,10^. This ecological flexibility could explain its hardiness and wide geographical distribution spanning four continents. Interspecies hybridization with closely related clariids has been shown to improve *C. gariepinus* environmental tolerance, manipulate sex ratios, and eventually increase growth performance, making it a highly efficient aquaculture fish^11^. As a result, the African catfish is considered an excellent biological model for studying amphibious traits (i.e., bimodal breathing) and terrestrial transition^12–14^. However, current genomics data has primarily focused on phylogenetic and domestication studies^15–17^, as well as on sex-chromosome and karyotype evolution utilizing a limited panel of molecular markers^18–20^. *Clarias gariepinus* genome is made up of 2*n* = 2*x* = 56 chromosomes (18 m + 20 sm + 18 st/a) with a variously described fundamental number (NF) between 88 and 110^21^. Its chromosome system has historically been contentious. Previous findings suggested a XX/XY male heterogametic chromosomal system^22–25^, while others pointed to a ZZ/ZW female heterogametic sex determination system (SDS)^26,27^. However, recent NGS data has suggested that both systems coexist in *C. gariepinus*^18,19^. The coexistence of both SDSs is influenced by environmental and social factors and geographical habitat. For example, the ZZ/ZW system is indicated in African wild ecotypes^21,26^, XX/XY system is observed in some anthropogenically introduced populations in Europe and China^23,24^, and both systems were evidenced within the same population in Thailand^18,28^.

Limited genomic resources, including reference genomes, haplotypes information, and expression data, have hampered the validation of these SDSs. However, few genomic resources of related clariid species, such as the walking catfish (*Clarias batrachus*)^29^ and the Indian catfish (*Clarias magur*)^30^, are publicly available. Although they are only at the scaffold levels and highly fragmented with thousands of contigs, these assemblies provide valuable resources for comparative genomic analyses. However, more high-quality genome data is still needed to advance our understanding of the evolution and adaptation of airbreathing catfish to terrestrial habitats. Gold standard genomes, such as telomere-to-telomere (T2T) and fully phased genomes^31–35^, not only facilitate studies of sex chromosome evolution and allele-specific expression, but also provide promising tools for investigating biological mechanisms that shape the robustness and evolution of species. The assembly of the T2T genome aims to build a complete and accurate representation of the chromosome from one telomere to the other^36^. This includes achieving end-to-end continuity and accurately resolving repetitive regions and structural variations. Identification of alleles that are collocated on the same chromosome is known as haplotype phasing. A fully phased genome assembly is one in which the two haplotypes (maternal and paternal) have been separated and assigned to their respective chromosomal sequences^37^. This means that each genomic region is related to a specific haplotype, allowing precise determination of allelic variants and specific haplotype information. Fully phased assemblies are especially useful for examining genetic variations, population genetic studies, and the inheritance of specific traits^38^. Fully phased assemblies can also improve the accuracy of genomic selection methods, which are increasingly being adopted in aquaculture breeding programs.

Here, we performed whole genome sequencing and assembly of *C. gariepinus* using HiFi PacBio, Oxford Nanopore Technologies (ONT) and Hi-C long-range phasing information. We obtained a near-T2T genome assembly of the African catfish. Our results provide a critical genomic basis for functional investigation of the molecular mechanisms underlying clariid evolution and their transition out of the water, with potential commercial and ecological implications.

## Methods

### Ethics Statement

All procedures involving the handling and treatment of the animals used in this study were approved by the Landesamt für Landwirtschaft, Lebensmittelsicherheitund Fischerei Mecklenburg-Vorpommern - Veterinärdienste und Landwirtschaft. The study was conducted in accordance with the local legislation and institutional requirements.

### Sample collection and DNA extraction

Tissue samples, including muscle, liver, and gonads, were collected from an adult male African catfish (approximately one year old) at the Experimental Aquaculture Facility of the Research Institute for Farm Animal Biology (Dummertorf, Germany). Before tissue collection, the fish was euthanized by immersing it in an overdose of 2-phenoxyethanol (50 mg/L) for 15 minutes, followed by a bleed cut at the head and posterior spinal cord. The tissue samples were immediately frozen in liquid nitrogen and stored at  − 80° C. Genomic DNA was extracted using the DNeasy Blood & Tissue Kit (Qiagen), following the manufacturer’s standard protocols. Library preparation strategies were tailored to the sequencing technologies used in this study.

### Libraries preparation and genome sequencing

**Fig. 1:**
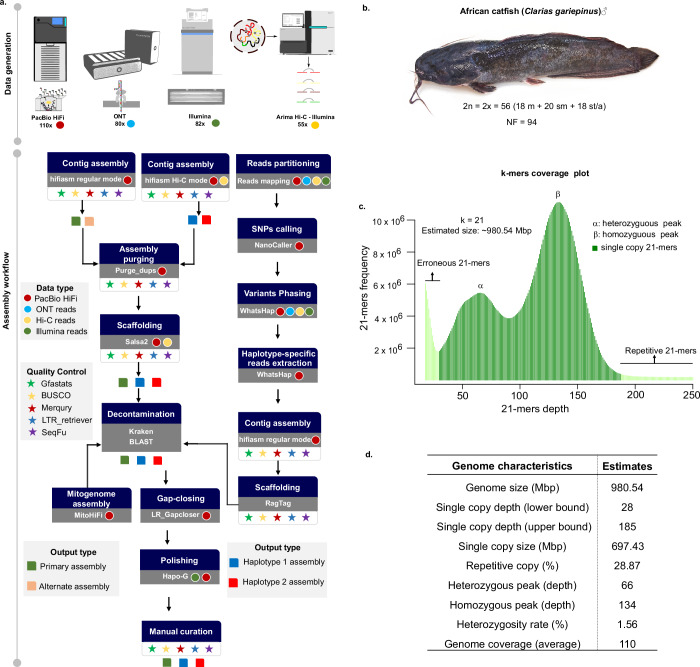
Haplotype-resolved genome assembly workflow of *Clarias gariepinus* and genome survey analysis. (**a**) The workflow was developed to build a haplotype-resolved genome assembly of the African catfish. Generated genomic sequencing data include Illumina paired-end 150, PacBio’s long high-fidelity (HiFi) reads, Oxford Nanopore (ONT) ultra-long reads and Hi-C data. A primary assembly and two haplotype-resolved assemblies were obtained using three assembly modes that combined different data types; (**b**) The African catfish specimen whose genome was sequenced in this study with the chromosome number for male individuals: A diploid genome with 18 metacentric (m), 20 submetacentric (sm), and 18 subtelomeric/acrocentric (st/a) chromosomes. NF is the fundamental number indicating the total number of chromosome arms; (**c**) *k*-mer frequency distribution of the diploid genome of the African catfish and its size estimate; (**d**) Preliminary genome characteristics estimated using *k*-mers analysis.

Genomic DNA (gDNA) sequencing data were generated using multiple platforms, including Oxford Nanopore (ONT) long reads, PacBio high-fidelity (HiFi) reads, Illumina paired-end reads, and paired-end Hi-C reads (Fig. 1a). Illumina short-insert (450 bp) libraries were prepared from liver tissues using the Illumina TruSeq Nano DNA Library Prep Kit and sequenced paired-end (PE150) on the Illumina Novaseq 6000 platform (Illumina, Inc., San Diego, CA, USA). Gonad tissues were used for ONT PromethION library preparation and sequencing, following the manufacturer’s guidelines (Oxford Nanopore Technologies). We sequenced a single flow cell on the PromethION instrument, generating 84 GB of data and a sequencing depth of approximately 80 × , with a maximum read length of 330 kb and an N50 of 32 kb. Liver and muscle tissues were pooled for HiFi library preparation and sequenced on the PacBio Sequel IIe platform (Pacific Biosciences of California, Inc.). Four SMRT cells were sequenced, producing approximately eight million CCS reads (141 Gb of data) with an N50 of 16 kb and an average base call accuracy exceeding 99.7%. A Hi-C library was generated using the Arima-HiC kit standard workflow (Arima Genomics, San Diego, CA, USA). All tissue samples were pooled and sequenced paired-end (PE150) on an Illumina HiSeq X Ten platform, generating 182 million read pairs, corresponding to approximately 55 × genome coverage (Table 1).

**Table 1:** Summary of sequencing data generated for the African catfish genome assembly.

Sequencing platform	Data type	Number of reads	Spanned length (Gbp)	Reads N50 (bp)	Coverage
Sequel IIe	HiFi/CCS	8,509,466	132.8	16,000	110x
PromethION 2	Nanopore	4,067,755	89.6	32,000	80x
NovaSeq 6000	Illumina PE-150	308,119,418	92.3	—	82x
HiSeq X Ten	Illumina PE-150 Hi-C	181,719,601	27.5	—	55x

### Genome survey analysis

To estimate the preliminary properties of the African catfish genome, we performed a genome-wide *k*-mer analysis using the k-mer Analysis Toolkit (KAT) (v2.2.0)^39^, based on high-quality genomic HiFi reads. Low-frequency *k*-mers (*depth* < 19) were filtered out. The 21-mer analysis revealed an estimated genome size of approximately 980 Mbp, a relatively high heterozygosity rate of 1.56%, and an expected repetitive sequence content of around 46% (Fig. 1c–d). The heterozygosity rate was calculated as (Number of distinct *k*-mers / Total number of *k*-mers) / 2. The 21-mer spectra histogram illustrates the high heterozygosity between both haplotypes, with homozygous regions consisting mainly of 2-copy *k*-mers and heterozygous regions consisting mostly of 1-copy *k*-mers, as expected from a diploid genome. These genomic properties were rendered using ggplot2 in R (Fig. 1).

### Haplotype-resolved chromosome-scale assemblies

**Fig. 2:**
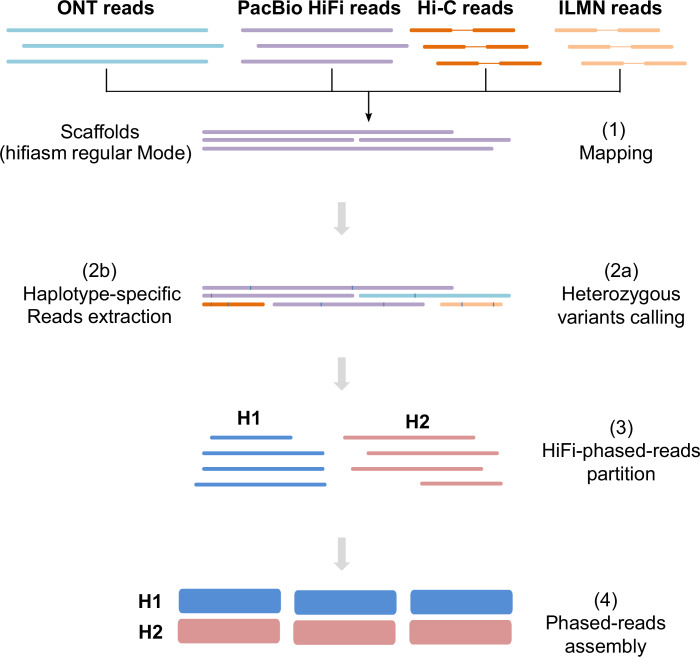
Reads partitioning (binning) assembly approach. The primary assembly obtained in Hifiasm regular mode was used as a reference. After aligning read data from ONT, PacBio HiFi, Hi-C, and Illumina to reference (1), heterozygous variants were called (2a), and haplotype-specific reads were extracted using WhatsHap (2b). Partitioned reads (3) were then *de novo* assembled into two distinct genome assemblies, one for each haplotype (4).

To construct the haplotype-resolved, chromosome-scale assemblies of the African catfish genome, we employed three strategies using the hifiasm assembler (v.0.16.1)^37^: regular mode for a primary assembly (Prim) and an alternate assembly (Alt), and a HiFi+Hi-C mode for producing two haploid assemblies (Hap1 and Hap2), representing the diploid genome’s parental haplotypes. We used the haplotype-resolved assembler hifiasm (v.0.16.1) in regular mode (i.e., without Hi-C data) with default parameters to build a contig-level primary and alternate assembly using clean PacBio HiFi reads. Additionally, a combination of HiFi and PE Hi-C reads was used in hifiasm to generate a set of two haplotype-resolved, phased contig-level (haploid) assemblies (i.e., hifiasm Hi-C mode). With purge_dups ((v1.2.6)^40^, we identified and removed contigs corresponding to haplotypic duplications, false duplications, sequence overlaps, and repeats. The phased contigs were scaffolded into chromosomes using a modified Arima Genomics Hi-C mapping pipeline and SALSA2 (v2.3)^41^. Subsequently, we aligned all generated reads with the primary assembly for heterozygous SNPs calling and reading binning into haplotype-specific datasets (read partitioning mode). Haplotype phasing was achieved using WhatsHap (v1.4)^42^, leveraging data from multiple sequencing platforms. The haplotype-specific reads were then independently assembled to produce high-quality haploid assemblies (Fig. 2). To check for putative contaminations, contigs were searched against all RefSeq microbial genomes using Kraken2^43^. In addition, a megaBLAST search was performed on RefSeq non-animal chromosome level assemblies, requiring the e-value ≤10^−5^ and the sequence identity ≥98%. We applied LR_Gapcloser^44^ with clean HiFi reads to fill unresolved gaps in the Prim assembly. The Hi-C contact maps were visually inspected, and manual curation was applied. The Hapo-G pipeline^45^ was used with default parameters to polish the Prim assembly using PacBio HiFi reads.

Following QC filtering and duplicate removal, the phased contig-level assembly of the African catfish genome yielded 58, 142, and 212 sequences with N50 values of 33.71 Mb, 32.12 Mb and 19.53 Mb for Primary, Haplotype-1 and Haplotype-2, respectively. As confirmed later by scaffolding with Hi-C data, more than half (*n* = 34) of the 58 primary contigs already represented complete chromosome arms or full-length chromosome arms. Primary assembly chromosomes were sorted and numbered by decreasing size. Chromosome sizes ranged from 52 Mbp (Chr 1) to 21 Mbp (Chr 28), with a median length of 32.3 Mbp. The high heterozygosity rate (1.56%) of the African catfish genome may have facilitated this successful haplotype separation, as it has previously been shown that a higher heterozygosity rate aids efficient genome unzipping^46^. The evolution of the assembly metrics after each processing stage is summarized in Supplement Tables S1–S3

### Genome annotation

The three assemblies were independently annotated to avoid a skewed comparison. The methods described here were used to annotate genes and repeats in haplotypes and primary assemblies. RepeatModeler (v2.0.3)^47^ was used to analyze and predict repeat sequences and dependencies such as TRF, RECON, and RepeatScout. Using MITE Tracker^48^, we identified miniature inverted-repeat transposable elements (MITEs). GenomeTools^49^ and LTR_Retriever (v2.9.0)^50^ were used to analyze full-length LTRs. Furthermore, we retrieved all teleost-specific transposable elements (TEs) from FishTEDB^51^, a curated database of TEs identified in complete fish genomes. We used cd-hit (v4.8.1)^52^ to cluster repeat elements with identities greater than 98%. Repeatmasker (v4.1.3)^53^ was used to mask the genome with the final custom non-redundant set of repeats.

Protein-coding genes in the *Clarias gariepinus* genome were annotated using ab initio, homology, and transcriptome-based methods. For homology-based annotation, high-quality protein sequences from UniProt and nine related catfish species were aligned with our African catfish assemblies using TBLASTN, applying an e-value cut-off of 1e-10 and a minimum identity threshold of 80%. The highest scoring alignments were used for the predictions of the gene model with miniprot (v0.13)^54^. For transcriptome-based predictions, we utilized quality-filtered RNA-Seq reads from the Sequence Read Archive (SRA) (BioProject-Accession: PRJNA487132), mapped using HISAT2 (v2.2.1)^55^, and transcripts assembled with StringTie2 (v2.2.0)^56^. *Ab initio* predictions integrated Augustus (v3.4.0)^57^, GeneMark-EP^58^, Genscan^59^, and GlimmerHMM^60^, with RNA-Seq data aiding in model training. A consensus gene set was generated using the funannotate pipeline (v1.8.13)^61^, filtering out genes lacking start or stop codons, containing in-frame stop codons, or shorter than 180 nt. Genes with high similarity to transposable elements were also excluded. Non-coding RNA genes were identified, including tRNAs with tRNAscan-SE^62^, ribosomal RNAs with RNAmmer^63^, and microRNAs using the miRDeep2 pipeline^64^ and miRBASE references^65^. Functional annotation of protein-coding genes was achieved using BLAST to align predicted protein sequences to RefSeq non-redundant proteins (NR), nucleotides (NT), and UniProtKB/Swiss-Prot databases. eggNOG-mapper (v2.1.9)^66^ and Interproscan (v5.56-89.0)^67^ were used to query BLAST top hits (query_coverage  > 60%, identity_score  > 80%) to obtain Gene Ontology (GO) annotations and gene names by ortholog transfer.

**Fig. 3:**
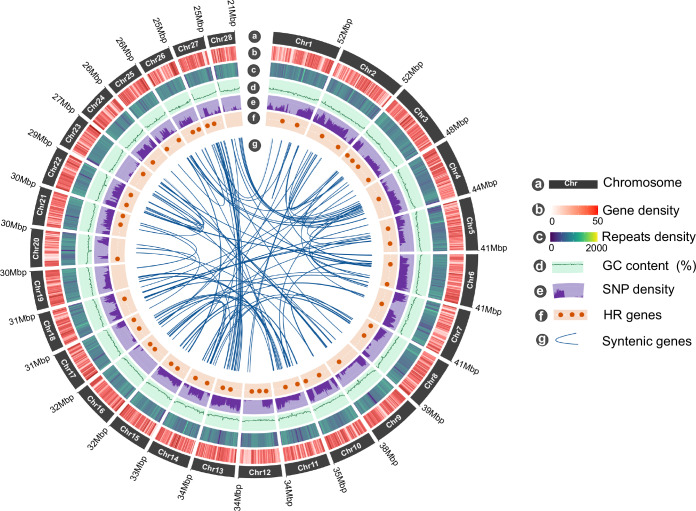
Genomic features of *Clarias gariepinus*. From the outer to the inner circle: (**a**) Length of the 28 diploid chromosomes (Mb); (**b**) Chromosome-wide gene density per non-overlapping 500 kb windows; (**c**) Repeats density in non-overlapping 500 kb windows; (**d**) GC content; (**e**) Distribution of heterozygous SNPs density; (**f**) Chromosomal loci of hypoxia-responsive (HR) genes predicted in the *C. gariepinus* genome; (**g**) The inner curve lines indicate syntenic gene pairs identified between *C. gariepinus* chromosomes.

In the primary assembly, 25,655 protein-coding gene models were predicted. The haploid Hap1 and Hap2 assemblies yielded slightly fewer predicted genes, with 23,577 and 24,223, respectively (Table 2). Approximately 200 genes predicted in the primary assembly were completely absent from the Hap1 and Hap2 assemblies. The primary assembly consistently resulted in a more comprehensive functional annotation, which is expected given that the diploid assembly includes both haplotypes and a complete representation of the genome structure. Overall, 87.80% of all high-quality proteins in the primary assembly and both haplotypes were assigned a functional annotation in at least one of the databases searched by sequence homology or ortholog mapping (Table 2). Repetitive sequences constituted 43.94% of the *C. gariepinus* genome, which roughly corresponded to the estimated repeat content of 46% based on k-mer analysis. Unlike that found in other catfish species, including *Clarias magur* (43.72%)^30^, *Clarias macrocephalus* (38.28%)^68^, *Pangasianodon hypophthalmus*(42.10%)^69^, and *Hemibagrus wyckioides* (40.12%)^70^. However, it is higher than in *Clarias batrachus* (30.30%)^29^, which has a smaller genome size (821.85 Mb). Interspersed repeats constitute the most abundant class of repetitive elements (46%), while retroelements and DNA transposons account for only 12% and 6% of the repeatome, respectively. The distribution of genes and repeats on chromosomes followed the typical pattern observed in vertebrate genomes, with higher gene densities in GC-rich regions and lower gene densities in repeat-rich distal and pericentromeric regions (Fig. 3).

**Table 2:** Summary of assembly metric of the *Clarias gariepinus* genome, including the primary (Prim), haplotype-1 (Hap1) and haplotype-2 (Hap2).

Category	Quality Metrics	Primary	Haplotype-1	Haplotype-2
General	Total assembly size (Mb)	969.67	968.90	954.25
	GC content	39.0	38.98	38.93
	Repeat content (%)	43.94	44.07	43.29
Continuity	No. Contigs	47	142	212
	Contig N50 (Mb)	33.71	32.12	19.53
	No. Scaffolds	47	135	175
	Scaffolds N50 (Mb)	33.71	34.0	33.18
	Scaffold L50	12	12	12
	Number of gaps	0	180	115
	% Unplaced sequences (Mbp)	1.01 (12.69)	1.70 (16.5)	2.63 (25.12)
	% Gapless length	100	99.99	98.54
Base accuracy	QV	41.86	38.14	39.39
Structural accuracy	k-mer completeness (%)	98.32	83.61	81.93
	Concondantly mapped PE reads (%)	96.75	96.69	97.81
	BUSCO duplicate (%)	0.55	0.58	1.26
	BUSCO missing (%)	0.70	0.58	0.99
	Reliably phased blocks (%)	—	96.87	94.00
Functional completeness	Protein coding genes	25,655	23,577	24,223
	BUSCO complete (%)	99.18	99.32	98.84
	BUSCO fragmented (%)	0.12	0.11	0.16
	NR annotation (%)	87.80	86.17	87.00
	Swissprot/Uniprot annotation (%)	68.23	63.12	64.45
	Transcripts alignment rate (%)	95.52	94.61	94.09

## Data Records

All raw high-throughput sequencing data analyzed in this project are publicly accessible. This dataset includes various sequencing methodologies such as Illumina paired-end (PE), Hi-C, PacBio HiFi, and Oxford Nanopore Technologies (ONT) sequencing reads. These data can be found under Sequence Read Archive (SRA) Project accession number SRP365618^71^. The whole genome assemblies and annotations have been deposited in the DDBJ/ENA/GenBank databases. The accessions for these datasets are as follows: the primary assembly is available at GCA_024256425.2^72^, Haplotype-1 at GCA_024256435.1^73^, and Haplotype-2 at GCA_024256465.1^74^. The versions of these assemblies described in this paper are GCA_024256425.2 for the primary assembly, GCA_024256435.1 for Haplotype-1, and GCA_024256465.1 for Haplotype-2. Additional resources on research design, and supplementary data, are available at Zenodo^75^.

## Technical Validation

### Structural and functional annotation

Approximately 99% of the assembled genome is spanned by 28 gapless chromosomes in the Prim assembly, while Hap1 and Hap2 contained only 0.1% and 1.44% unresolved nucleotides (gaps), respectively, mainly in repeat-rich genomic regions. The final haplotype-resolved assembly size for Prim, Hap1 and Hap2 is 969.72 Mb, 972.60 Mb, and 954.24 Mb, respectively. Only Hap2 dramatically increased the N50 metric from 19 Mb to more than 33 Mb at the scaffold level (Table 2). The Hap1 and Hap2 assemblies resulted in 23,577 and 24,223 predicted genes, respectively, showing about 200 genes absent compared to the primary assembly (Table 2). The primary assembly provided a more comprehensive gene annotation due to its complete genome representation. Functional annotation was achieved for 87.80% of the 73,455 high-quality proteins identified in all assemblies. The *C. gariepinus* genome exhibited a high repeat content of 43.94%, comparable to other species of catfish, and consisted predominantly of interspersed repeats (46%), with retroelements and DNA transposons that form 12% and 6% of the repeatome, respectively.

### Assembly and gene space completeness

To ensure the high quality and completeness of our haplotype-phased African catfish genome assembly, we conducted a comprehensive evaluation encompassing gene space completeness, full-length transcript coverage, read mappability, phasing accuracy, and genomic *k*-mer completeness. The BUSCO analysis showed a completeness of 99.10%, with comparable results between the haplotypes and the primary assemblies. Given that only 0.7% of the expected universal orthologs were missing, we conclude that the gene space covered by our genome assembly is nearly complete (Table 2). Additionally, approximately 92% of *C. gariepinus* transcripts were successfully mapped to our assemblies, with over 90% coverage and more than 90% identity, demonstrating high functional completeness. We also evaluated structural accuracy by mapping genomic reads to our assemblies and found that over 96.69% of raw paired-end (PE) reads were concordantly aligned. The alignment rates for ONT, HiFi, and Hi-C reads in the primary assembly were 99.91%, 99.95%, and 100%, respectively. The mapping rates for Hap1 and Hap2 assemblies were also above 99% (Table 3).

**Table 3:** Summary statistics on genomic reads mapping on Haplotype-1 (Hap1), Haplotype-2 (Hap2) and Primaray (Prim) assemblies.

Data	Hap1 (%)	Hap2 (%)	Prim (%)
ONT	99.91	99.92	99.91
HiFi	99.52	99.36	99.95
Hi-C PE	99.95	99.98	100
Illumina PE	98.89	99.22	99.03
Illumina PE (properly paired)	96.69	97.81	96.75
Illumina PE mRNA-Seq	90.61	89.09	92.52

### Phasing and structural validation

**Fig. 4:**
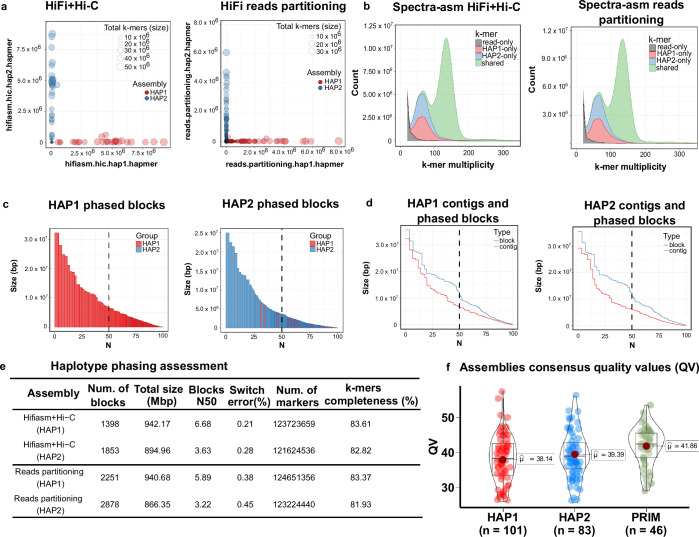
QC plots for evaluating haplotype phasing accuracy, genome contiguity and completeness. (**a**) Hap-mers blob plot of the Hifi+Hi-C (left) and HiFi reads partitioning assembly (right). Red blobs represent HAP1-specific *k*-mers, while blue blobs are the HAP2-specific *k*-mers. Blob size is proportional to chromosome size. A well-phased assembly should have orthogonal hapmers (e.g. HAP1 and HAP2 lie along the axes, respectively). Both assemblies show nearly no haplotype mixture; (**b**) Spectra-asm plot of HiFi+Hi-C (left) and Reads partitioning (right) assemblies. The 1-copy k-mers representing the heterozygous alleles are specific to each haplotype assembly (HAP1 and HAP2), and the 2-copy k-mers, which are only found in the diploid genome, are shared by both assemblies (green). There is no discernible difference between the two assembly approaches. Low-copy *k*-mers (depth < 18) arising from contamination or sequencing errors were removed from the visualization; (**c**) Phased blocks N* plots of HAP1 (left) and HAP2 (right) assembly, sorted by size. The X-axis represents the percentage of the assembly size (*) covered by phased blocks of this size or larger (Y-axis). Blocks from the incorrect haplotype (haplotype switches) are tiny and almost absent in the other haplotype. In both haplotypes, more than 75% of the assembly is spanned by phased blocks larger than 1 Mbp; (**d**) Phase block and contig N* plots showing the relative continuity of HAP1 (left) and HAP2 (right); (**e**) Statistics for haplotype phasing with switch errors and phased blocks allowing up to 100 switches within 20 kbp; (**f**) The average consensus quality (QV) distribution for each assembly. Each dot represents a scaffold in the associated assembly.

Merqury evaluated the quality of the assembly by analyzing the consistency and precision of the phasing of the haplotype using *k*-mers specific haplotypes. Ideally, these *k*-mers should be entirely distinct between haplotypes in a perfectly phased assembly. Our data indicated high orthogonality between Hap1 and Hap2 with minimal haplotype switches and almost no contamination (Fig. 4a). Homozygous *k*-mers shared in the 2-copy peak and distinct heterozygous *k*-mers appearing in the 1-copy peak demonstrated effective haplotype separation (Fig. 4b). However, this method only estimates how well each haplotype recovers heterozygous k-mers because only orthogonal reads (e.g. parental) can independently determine the factual phasing accuracy. Although haplotype-specific reads can simulate parental reads, they will still miss a few true heterozygous parental *k*-mers due to sequencing bias or sequencing errors.

**Fig. 5:**
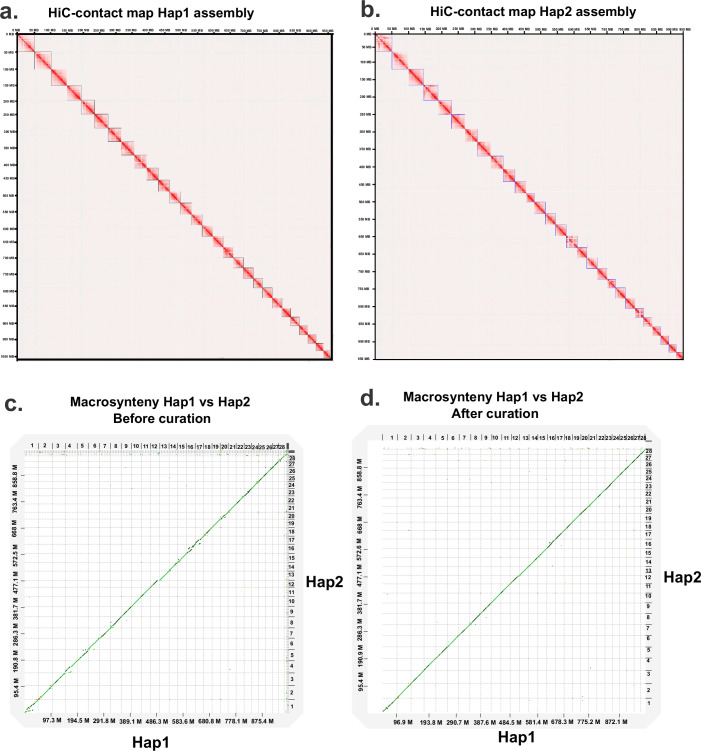
Genome-wide contact map of the curated Hap1 and Hap2 assemblies and macrosynteny between haplotypes. Part (**a**) and (**b**) depict the genome-wide Hi-C contact map of haplotype-1 (Hap1) and haplotype-2 (Hap2), respectively. Squares on the diagonal represents chromosomes in decreasing order (Chr1 in upper left corner). The Hi-C heatmap was generated at a high resolution of 50 kbp. Warmer colors indicate more frequent interactions between genomic regions, while cooler colors denote less frequent interactions; (**c**) presents a macrosynteny plot comparing the two haplotype assemblies before curation; (**d**) shows the same comparison after curation, highlighting the improved alignment and structural consistency achieved through the curation process.

To validate the structural consistency of the assembly, we generated genome-wide Hi-C contact maps for the Hap1 and Hap2 assemblies. The Hi-C contact map for the curated Hap1 assembly (Fig. 5a) demonstrates the interaction frequencies between different genomic regions, indicating a high-quality assembly with clear chromosomal boundaries. Similarly, the Hi-C contact map for the curated Hap2 assembly (Fig. 5b) shows comparable interaction patterns, confirming the structural integrity and accuracy of the pahased assemblies. We observed size differences and structural variations (SVs) between haplotypes in the African catfish genome, particularly in Chr1 and Chr16. Imbalanced haplotype assemblies, particularly in species exhibiting high heterogeneity in sex chromosome sizes, are not uncommon. Such size heterogeneity may result in observable discrepancies in the sizes of haplotype assemblies, especially within regions specific to sex loci. After manual curation, we achieved much fewer discrepancies between haplotypes compared to before manual curation (Fig. 5c–d), suggesting that manual curation greatly improved the quality of both haplotype assemblies. The proportion of base pairs affected by SV decreased from 0.36% (before curation) to 0.12% (after curation). However, even manual curation could not fix a large inversion in Chr1 and a translocation in Chr16 (Fig. 5d), suggesting a potentially data-induced inconsistency. More data and experiments are needed to verify these structural variations. As suggested by the Hi-C contact map and the synteny map, the two haplotype assemblies showed high consistency in sequence order and orientation.

### Validation of chromosomal telomeres

**Fig. 6:**
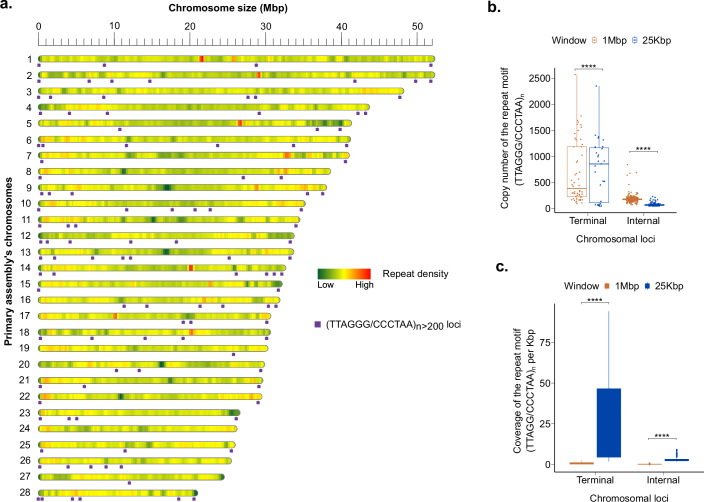
Genome-wide telomere portrait of *Clarias gariepinus*. (**a**) The purple boxes are chromosomal loci of the tandemly repeated telomeric motif (*TTAGGG*/*CCCTAA*)_*n*>200_ in the Primary assembly. Only telomeric repeats with a minimum size of 1200 bp are shown. The heatmap shows the chromosome-wide repeat density in non-overlapping 500 kbp windows; (**b**) Boxplots show the copy number distribution of the telomeric repeat motifs (*TTAGGG*/*CCCTAA*)_*n*>45_ in Terminal and Internal 25 kbp and 1 Mbp window. **** are statistical significance levels of the T-test ($p \mbox{-} value < 0.0001\left.\right)$); (**c**) Boxplots depict the density of (*TTAGGG*/*CCCTAA*)_*n*>45_ motif per 1000 bp in Terminal and Internal 25 kbp and 1 Mbp window.

Using the telomere identification toolkit (tidk)^76^, we scanned *C. gariepinus* genome for terminal telomeric repeats ${(5{\prime} -{\rm{TTAGGG}}-3{\prime} )}_{n}$ with a minimum length of 270 bp (*n* = 45) in 25 kb windows of chromosomal termini. To be termed ’terminal telomeric repeats’, we required the motif (*TTAGGG*/*CCCTAA*)_*n*_ to exhibit the highest density per 25 kb in the terminal 25 kb windows compared to internal 25 kb windows. All non-terminal telomeric repeats are referred to as internal or interstitial telomeric sequences (ITS). Our study did not only detect both terminal telomeres in 22 of 28 chromosomes (Fig. 6a), but also several ITS with high copy numbers (*n* > 200), mainly located in the pericentromeric regions and along the nucleolar organizer regions (NORs). These interstitial telomeric sequences have previously been reported as relics of genome rearrangements in some vertebrate species.

The absence of high-density terminal telomeric signals at both ends of few chromosomes (*n* = 5) is not necessarily due to the poor assembly of these regions. Telomeres could also be lost or gradually shortened on these chromosomes. The *C. garipinus* genome consists of nine subtelomeric/acrocentric (st/a) chromosomes. It has been established that st/a chromosomes have a very short p-arm and that the length of their telomeres is often shorter than that of other types of chromosome^77^. Extending the search window to 1 Mbp did not result in a significantly larger number of terminal telomeric repeats (*p*. *adjust* < 0.01). The 25 kbp terminal windows exhibited significantly larger telomere sizes and densities per kbp than terminal 1 Mbp windows (Fig. 6b–c). This observation suggests that the 25 kbp terminal windows captures the majority of full-length terminal telomeric repeats in our African catfish chromosomal assembly, consistent with previous findings indicating that the length of telomeric DNA in fish ranges from 2 to 25 kb^78–80^.

## Usage Note

The genome assemblies and associated datasets generated in this study provide valuable resources for researchers investigating the genetics and genomics of air-breathing catfish and related species. These data can be used to explore various biological questions, including the evolution of air-breathing, adaptation to terrestrial habitats, and the genetic basis of aquaculture traits. We recommend that researchers utilize the most recent version of the genome assembly and annotation files, as these will be periodically updated with new data.

## Supplementary information

Supplement Tables

## Data Availability

If specific parameters were not mentioned, all software and tools in this study were utilized with their default settings. Custom scripts and pipelines used in data analysis and to create figures are freely available at Zenedo^75^.
