# Supplementary material for: Haplotype-resolved and near-T2T genome assembly of the African catfish (Clarias gariepinus)
Source: Sci Data. 2024 Oct 7;11:1095. doi: 10.1038/s41597-024-03906-9 (PMC11458897; doi:10.1038/s41597-024-03906-9)
Supplement: Supplementary file 1 — Supplement Tables [file 41597_2024_3906_MOESM1_ESM.docx]

**Supplement Tables S1-S3:**

**Haplotype-resolved assembly of the African catfish (Clarias gariepinus) provides insights for semi-terrestrial adaptation of airbreathing catfishes**

**Supplement Table S1: Assembly metrics for each stage of the haplotype-1 (Hap1) assembly process.**

| Assembly Metrics  (Haplotype-1) | Prior to Purging Duplicates | After Purging Duplicates | After Scaffolding |
| --- | --- | --- | --- |
| Sum (bp) | 975,642,339 | 962,981,634 | 959,649,586 |
| Number of Contigs (n) | 374 | 138 | 119 |
| Average Length (bp) | 4,346,637.27 | 7,079,577.06 | 8,173,525.93 |
| Largest Contig | 51,836,041 | 51,775,064 | 51,775,064 |
| N50 | 19,535,869 | 33,967,633 | 34,021,760 |
| N60 | 16,105,069 | 33,010,436 | 31,826,834 |
| N70 | 12,094,094 | 30,681,493 | 30,693,607 |
| N80 | 8,696,350 | 29,291,423 | 29,569,184 |
| N90 | 3,252,443 | 25,500,161 | 26,084,726 |
| N100 | 1,685 | 11,440 | 10,904 |
| N_count | 0 | 0 | 16,693 |
| Gaps | 0 | 0 | 182 |

**Supplement Table S2: Assembly metrics for each stage of the haplotype-2 (Hap2) assembly process.**

| Assembly Metrics  (Haplotype-2) | Prior to Purging Duplicates | After Purging Duplicates | After Scaffolding |
| --- | --- | --- | --- |
| Sum (bp) | 9,861,947,652 | 956,051,486 | 954,244,515 |
| Number of Contigs (n) | 725 | 215 | 98 |
| Average Length (bp) | 2,233,760.72 | 9,657,085.72 | 9,737,188.93 |
| Largest Contig | 51,514,250 | 50,534,992 | 50,467,679 |
| N50 | 19,360,100 | 19,220,744 | 33,187,444 |
| N60 | 16,488,403 | 31,639,599 | 31,492,657 |
| N70 | 12,289,679 | 29,998,812 | 29,978,869 |
| N80 | 8,122,215 | 25,817,995 | 25,739,244 |
| N90 | 2,834,834 | 25,004,727 | 24,914,970 |
| N100 | 1,685 | 6,098 | 10,508 |
| N_count | 0 | 0 | 140,152 |
| Gaps | 0 | 0 | 115 |

**Supplement Table S3: Assembly metrics for each stage of the primary (Prim) assembly process.**

| Assembly Metrics  (Primary) | Prior to Purging Duplicates | After Purging Duplicates | After Scaffolding | After Gap Filling |
| --- | --- | --- | --- | --- |
| Sum (bp) | 975,107,719 | 970,894,268 | 970,899,868 | 969,620,887 |
| Number of Contigs (n) | 86 | 58 | 55 | 47 |
| Average Length (bp) | 11,338,461.85 | 14,936,834.89 | 17,652,713.96 | 19,790,222.18 |
| Largest Contig | 52,514,250 | 52,268,991 | 52,273,659 | 52,237,485 |
| N50 | 25,973,815 | 30,648,538 | 33,729,462 | 33,715,535 |
| N60 | 22,137,932 | 30,288,788 | 32,181,005 | 32,143,731 |
| N70 | 20,934,613 | 29,508,376 | 30,627,632 | 30,601,865 |
| N80 | 17,208,021 | 26,232,783 | 29,619,443 | 29,591,905 |
| N90 | 10,345,444 | 22,011,831 | 25,968,190 | 25,952,957 |
| N100 | 1,685 | 3,148 | 3,148 | 20,824 |
| N_count | 0 | 0 | 600 | 0 |
| Gaps | 0 | 0 | 6 | 0 |
